# Mayaro Virus in Wild Mammals, French Guiana

**DOI:** 10.3201/eid0910.030161

**Published:** 2003-10

**Authors:** Benoît de Thoisy, Jacques Gardon, Rosa Alba Salas, Jacques Morvan, Mirdad Kazanji

**Affiliations:** * Institut Pasteur de la Guyane, French Guiana; † Caribbean Epidemiology Center (CAREC), Port of Spain, Republic of Trinidad and Tobago

## Abstract

A serologic survey for Mayaro virus (*Alphavirus, Togaviridae*) in 28 wild nonflying forest mammal species in French Guiana showed a prevalence ranging from 0% to 52% and increasing with age. Species active during the day and those who spent time in trees were significantly more infected, results consistent with transmission implicating diurnal mosquitoes and continuous infectious pressure.

The Mayaro virus is a zoonotic Alphavirus (family *Togaviridae*) responsible for epidemics of febrile exanthematous illness in Latin America and Amazonia (*1*). Although the death rate is low, Mayaro fever is a major arboviral infection relevant to public health in rural populations, with an increasing incidence of human cases in the Amazonian basin following ecosystem disturbances (*2*). The activity of the Mayaro virus can be described as a constantly moving wave, transmitted among susceptible vertebrates by sylvatic *Culicidae* mosquitoes (*3*). *Haemagogus* mosquitoes are the main vectors; they are diurnal canopy dwellers common in high pristine rainforests (*4*). Several vertebrate hosts, mainly primates, rodents, and birds, are considered to be reservoirs, although their exact role in maintaining the virus is insufficiently understood (*2*). Previous serologic surveys have shown high prevalence rates in primates, but the virus has been isolated only from lizards and one marmoset (*5*,*6*); experimental inoculation of marmosets resulted in a short period of viremia, although the titer was nevertheless probably high enough to infect vectors (*4*). Human disease outbreaks could occur when birds or vectors introduce the virus into rural areas with high densities of both *Haemagogus* and potential reservoirs.

This emerging disease has recently been reported in French Guiana, a French administrative unit on the northern coast of South America (*6*). To investigate the diversity of the reservoir species, a serologic survey for Mayaro virus was conducted on 28 nonflying mammalian rainforest species, in a total of 579 animals; no previous surveys in wild vertebrate species has included so many samples. Investigations on antibody responses in potential hosts are important first steps for understanding viral dynamics (*7*). Since the infectious process plays a major role in wildlife ecology (*8*), we used Mayaro infection as a case study to investigate the correlation between ecologic and biologic patterns of potential hosts and their susceptibility to infection.

## The Study

Blood samples were collected in 1994–95 during a wildlife rescue operation at the Petit Saut hydroelectric dam site (4°55′ N, 53°05′ W), French Guiana (*9*). The overall habitat of all species was pristine high rainforest. Each serum sample was tested by hemagglutination inhibition (HI) for antibodies to Mayaro and Tonate (Venezualan equine encephalitis complex) viruses. Serum samples with titers >1:20 were confirmed by seroneutralization at a 1:20 dilution (*10*). Briefly, equal volumes of diluted serum were mixed with a Mayaro virus suspension containing 100 tissue culture infectious dose 50 per 0.1 mL. The reaction was incubated at 37°C for 1 h. As control of the test dose of virus, the working dilution was successively diluted 10-fold. One hundred microliters of the serum-virus mixture, control virus, and diluted serum was inoculated in duplicate in monolayers of Vero-E6 cell line and incubated at 37°C for 5 to 7 days. A positive reaction by neutralization was considered with the total inhibition of the cytopathic effect in the cell monolayer induced by Mayaro virus. Analysis of variance (p<0.05 considered significant) was used to study correlations between ecologic patterns and arcsine-transformed seropositivity of species (XlStat-Pro, Addinsoft, Paris, France). The following ecologic parameters were considered: vertical use of space (arboreal, terrestrial, and both); density (low <10 individual animals/km^2^; medium 10–20 individual animals, and high >20 individual animals/km^2^); rhythm of activity (strictly diurnal, strictly nocturnal, or both); and lifespan. Because lifespan data in the wild are not available for most species, we used last reproduction age as an indicator, and we classified lifespan as short (last reproduction age <5 y), intermediate (last reproduction age between 5 and 15 y) , and long (last reproduction age >15 y). The data used are summarized in Table 1.

**Table 1:** Ecologic data used in the multivariate analysis^a^

Species	Vertical use of space	Rhythm of activity	Lifespan	Density
*Choloepus didactylus*	A	ND	L	M
*Tamandua tetradactyla*	AT	ND	L	M
*Bradypus tridactylus*	A	ND	L	H
*Dasypus* spp*.*	T	N	I	H
*Myoprocta acouchy*	T	D	I	M
*Dasyprocta leporina*	T	D	I	H
*Agouti paca*	T	N	I	H
*Coendou melanurus*	A	N	I	M
*C*. *prehensilis*	A	N	I	H
*Echimys spp.*	A	N	I	M
*Proechimys* sp.	T	N	I	H
*Mazama* spp.	T	ND	I	L
*Tayassu tajacu*	T	D	I	M
*Potos flavus*	A	N	I	M
*Nasua* *nasua* & *Eira* *barbara*	AT	D	I	L
*Didelphis marsupialis*	AT	N	S	M
*Didelphis albiventris*	AT	N	S	M
*Caluromys philander*	AT	N	S	H
*Metachirus nudicaudatus*	T	N	S	M
*Caluromys philander*	AT	N	S	H
*Alouatta seniculus*	A	D	L	M
*Saimiri sciureus*	A	D	I	M
*Pithecia pithecia*	A	D	I	L
*Saguinus midas*	A	D	I	M

^a^Vertical use of space: A, arboreal, T, terrestrial, AT, both arboreal and terrestrial. Rhythm of activity: D, diurnal; N, nocturnal; ND, nocturnal and diurnal. Lifespan: S, short; I, intermediate; L, long (see text). Density: L, low; M, medium; H , high (see text).

All the serum samples were negative for Tonate virus, which suggests that no cross-reaction occurred with Mayaro virus. The possibility of cross-reaction with Una virus, a closely genetically related Togavirus, was not considered since Una is known only in subtropical areas (*11*) and open habitats (*12*,*13*). The seroprevalence rates of Mayaro virus, on the basis of seroneutralization confirmation, ranged from 0% to 52% and reached 80% in two species (Table 2). However, only a limited sample was available. Gender had no apparent effect on the frequency of infection. The prevalence of infection increases with age in howler monkeys (*14*). A similar pattern has been observed in sloths, with only adults found to be seropositive. On the basis of hematologic and biochemical data, the virus infection had no apparent effect on the animal’s health (*14*,*15*). Patterns of activity and vertical use of space were the two parameters with the greatest significant predictive value for positive serologic test results (p=0.03 and 0.01, respectively). The best analysis of variance model fitted these two variables (R^2^=0.45, p<0.02). Species that are active during daytime and arboreal or arboreal/terrestrial species were found to be more frequently infected than others primates of the *Cebidae* family (howlers, sakis, and squirrel monkeys); two-toed sloths had the highest prevalence. Prevalence rates were highly variable among species exhibiting only one of the explicative patterns; and no species that is strictly terrestrial and nocturnal, such as the four-eyed opossum, the spiny rat, and the paca, was infected (Figure).

**Table 2:** Mayaro virus seroprevalence in free-ranging non-flying mammals, French Guiana (seroneutralization assay)

Order	Species (n)	Seroprevalence (%)
Xenarthra	2-toed sloth, *Choloepus* *didactylus* (26)	27
	3-toed sloth, *Bradypus* *tridactylus* (29)	3
	Kappler armadillo, *Dasypus* *kappleri* (20)	0
	Nine-banded armadillo, *D. novemcinctus* (40)	10
	Collared anteater, *Tamandua* *tetradactyla* (26)	23
Marsupiala	Common opossum, *Didelphis marsupialis* (29)	3
	White-eared opossum, *D*. *albiventris* (19)	10
	Brown 4-eyed opossum, *Metachirus* *nudicaudatus* (19)	0
	Grey 4-eyed opossum, *Philander* *opossum* (27)	19
	Woolly opossum, *Caluromys* *philander* (5)	20
Rodentia	Acouchy, *Myoprocta acouchy* (29)	0
	Red-rumped agouti, *Dasyprocta* *leporina* (29)	17
	Brazilian porcupine, *Coendou* *prehensilis* (26)	11
	Black-tailed porcupine, *Coendou* *melanurus* (15)	13
	Paca, *Agouti* *paca* (17)	0
	Terrestrial spiny rat, *Proechimys* sp. (18)	5
	Arboreal spiny rat, *Echimys* spp. (21)	5
Carnivora	Kinkajou, *Potos* *flavus* (9)	11
	Coati, *Nasua* *nasua* and Tayra, *Eira* *barbara* (7)	11
Artiodactyla	Collared peccary, *Tayassu* *tajacu* (7)	0
	Brocket deers, *Mazama* spp. (10)	0
Primata	Red howler monkey, *Alouatta* *seniculus* (98)	52
	White-faced saki, *Pithecia* *pithecia* (5)	80
	Squirrel monkey, *Saimiri* *sciureus* (6)	67
	Golden-handed tamarin, *Saguinus* *midas* (42)	19

**Figure:**
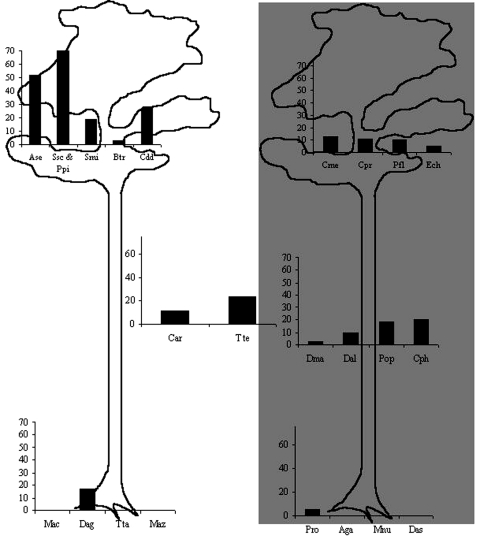
Mayaro virus seroprevalence rates in Neotropical mammal species, according to 1) activity period of animals (diurnal species on the left, nocturnal species on the right, diurnal and nocturnal species in the center); 2) vertical use of space (terrestrial, arboreal, or both). **Ase, *Alouatta* *seniculus*; Smi , *Saguinus* *midas*; Ssc, *Saimiri* *sciureus*; Ppi, *Pithecia* *pithecia*; Btr, *Bradypus* *tridactylus*; Cdd, *Choloepus* *didactylus*; Tte, *Tamandua* *tetradactyla*; Car, carnivores (*Nasua* *nasua* and *Eira* *barbara*); Cme, *Coendou* *melanurus*, Cpr, *Coendou* *prehensilis*; Ech, *Echymis* spp.; Pfl, *Potos* *flavus*; Dma, *Didelphis* *marsupialis*, Dal, *Didelphis* *albiventris*; Pop, *Philander* *opossum*; Cph, *Caluromys* *philander*; Mac, *Myoprocta* *acouchy*; Dag, *Dasyprocta* *leporina*; Tta, *Tayassu* *tajacu*; Pro, *Proechimys* sp.; Apa, *Agouti* *paca*; Mnu, *Metachirus* *nudicaudatus*; Das, *Dasypus* spp., Maz, *Mazama* spp.**

## Discussion

Ecological dynamics are often not included in the epidemiology of Amazonian diseases; the diversity of arthropods and vertebrates and their ecological conditions and the difficulty in obtaining samples have resulted in limited understanding of arboviral infection patterns. This survey of the Mayaro virus in the French Guiana rainforest was based on a large number of individual animals and a wide variety of nonflying mammalian species with different ecologic habits. The survey corroborated previously reported epidemiologic patterns while providing some additional features. No bird, bat, or reptile species was included in the survey, since the wildlife rescue operation to collect samples was not focused on such species. But although limited to nonflying mammals, the multivariate analysis shows that arboreal or diurnal species are infected significantly more frequently than others. Thus, most hosts are bitten while in the upper forest layer and when foraging at the same hours as the vectors. The previously reported infection of sloths and howler monkeys (*5*) is explained by our findings, as their way of life comprises the two factors found to be linked to infection. Mayaro infection has also been reported in agoutis (*5*): infection of these strictly terrestrial diurnal species may reflect the ecological plasticity of the vector or may indicate that other mosquitoes, with different ecologic patterns, are implicated in transmission. The fact that lifespan and density do not contribute to the variation in seroprevalence may reflect the ecological ubiquity of the vectors and the fact that the virus circulates easily in both the vectors and the host populations (*16*). Increasing seroprevalence with age has been also described in sloths with St. Louis encephalitis virus (*17*) and could also be related to constant activity of the arbovirus and infectious pressure. Comparative seroprevalence surveys in areas with different host diversity and density would be of interest.

Some strong differences were observed between species with ecologic patterns favorable to infection. For example, the two-toed sloths and large primates were more frequently infected than three-toed sloths, carnivores, and tamarins. These differences may be linked to microhabitat use, behavioral patterns, or specific sensitivity. Although the geometric HI mean titer and seroprevalence were not significantly linked (i.e., species with greater exposure appeared to have lower titers), this finding could be related to continual exposure to virus infection risk and a latent or chronic infection (*18*). On the contrary, animals from species less exposed to infection (kinkajous, four-eyed opossums, arboreal porcupines) showed higher titers, probably because of accidental infections resulting in intense immunologic response. However, more data on virus isolation and experimental infections are necessary to confirm those assumptions.

Information on the incidence of disease and the pathogenicity of infectious agents in wildlife is still limited. Although the susceptibility of a host species remains speculative when the agent has not been isolated, a positive antibody response shows that a specific antigen, or a serologically closely related antigen, is or was present, and that the infected species has been exposed and has responded. Serologic investigations in free-ranging species are often limited to transversal surveys in several animals at a single time. The levels and distribution of seropositivity and titers can, however, be used for a better understanding of both virus dynamics and host susceptibility. In infections with alphaviruses, the viremia is intense but short (*7*), but the diversity of the potential reservoirs may compensate for this short period. When the virus is introduced into a new area, many individual animals can be infected rapidly. The low individual excretion rate is compensated for by the number of animals possibly affected by the virus. This viral strategy is completely opposite to that of the *Arenaviridae*, where the reservoirs are much less diverse (usually a single species), but the hosts occur at high density and viral excretion is long-lasting (*19*). Other arboviruses with an ecologic niche similar to that of the Mayaro virus may also have a wide diversity of potential reservoirs. The Mayaro virus is active mainly in forests, but *Haemagogus* spp. can fly over large areas. Howler monkeys, tamarins, squirrel monkeys, and agoutis are also still common in the vicinity of the main cities of French Guiana, and periurban species may also be infected and act as reservoirs. Moreover, alphaviruses are prone to be hosted by a large range of vectors (*3*) and the risk for new epidemic patterns, comparable to the emergence of periurban Chikungunya cycle in Africa, should be considered (*1*). Demographic pressure is resulting in increasing human contact with forest habitats and fauna; the predictable health consequences of this evolution have been emphasized in parasites (*20*) and may also be applicable in arboviruses.
